# Central urocortin 3 and type 2 corticotropin-releasing factor receptor in the regulation of energy homeostasis: critical involvement of the ventromedial hypothalamus

**DOI:** 10.3389/fendo.2012.00180

**Published:** 2013-01-07

**Authors:** Peilin Chen, Christine Van Hover, Daniel Lindberg, Chien Li

**Affiliations:** ^1^Department of Pharmacology, University of Virginia Health SystemCharlottesville, VA, USA; ^2^Department of Neuroscience, University of Virginia Health SystemCharlottesville, VA, USA

**Keywords:** CRF, Ucn 3, VMH, energy balance, feeding, glucose

## Abstract

The vital role of the corticotropin-releasing factor (CRF) peptide family in the brain in coordinating response to stress has been extensively documented. The effects of CRF are mediated by two G-protein-coupled receptors, type 1 and type 2 CRF receptors (CRF_1_ and CRF_2_). While the functional role of CRF_1_ in hormonal and behavioral adaptation to stress is well-known, the physiological significance of CRF_2_ remains to be fully appreciated. Accumulating evidence has indicated that CRF_2_ and its selective ligands including urocortin 3 (Ucn 3) are important molecular mediators in regulating energy balance. Ucn 3 is the latest addition of the CRF family of peptides and is highly selective for CRF_2_. Recent studies have shown that central Ucn 3 is important in a number of homeostatic functions including suppression of feeding, regulation of blood glucose levels, and thermoregulation, thus reinforcing the functional role of central CRF_2_ in metabolic regulation. The brain loci that mediate the central effects of Ucn 3 remain to be fully determined. Anatomical and functional evidence has suggested that the ventromedial hypothalamus (VMH), where CRF_2_ is prominently expressed, appears to be instrumental in mediating the effects of Ucn 3 on energy balance, permitting Ucn 3-mediated modulation of feeding and glycemic control. Thus, the Ucn 3-VMH CRF_2_ system is an important neural pathway in the regulation of energy homeostasis and potentially plays a critical role in energy adaptation in response to metabolic perturbations and stress to maintain energy balance.

## INTRODUCTION

Corticotropin-releasing factor (CRF) was discovered in 1981 by Vale and colleagues at the Salk Institute (Vale et al., 1981). Its existence had been hypothesized for many years prior to the 1981 paper characterizing the amino acid sequence of the peptide. Since then, the importance of CRF family of peptides and receptors in stress has steadily emerged. Extensive investigation has demonstrated that CRF is critical in regulating the hypothalamic-pituitary-adrenal (HPA) axis and in integrating endocrine, autonomic, and behavioral responses to stressors (Perrin and Vale, 1999).

In addition to CRF, additional members of the CRF peptide family including urocortins (Ucns) 1, 2, and 3 have been identified in mammals, including humans (Vaughan et al., 1995; Hsu and Hsueh, 2001; Lewis et al., 2001; Reyes et al., 2001). Accumulating evidence suggests that the central actions of Ucn peptides may account for some stress-related effects originally attributed to CRF (Bale and Vale, 2004; Hashimoto et al., 2004; Venihaki et al., 2004; Jamieson et al., 2006; Kuperman et al., 2010). For example, Ucn 1 appears to be involved in the later stage of the stress response and adaptation to stress, while Ucns 2 and 3 may be involved in attenuating the stress response (Ryabinin et al., 2012).

Additionally, the Ucns have been shown to be involved in various physiological regulations including energy balance, cardiovascular function, and behavioral modulation. Ucn 1, 2, and 3 all suppress feeding (Spina et al., 1996; Hashimoto et al., 2004; Fekete et al., 2007), and deficiency in Ucn 2 improves glucose and insulin homeostasis (Chen et al., 2006). Ucn 1 and 2 decrease cardiac output and heart rate, and may be protective against ischemia (Latchman, 2002; Bale et al., 2004; Hashimoto et al., 2004). Behaviorally, Ucn 1 plays a critical role in anxiety-like and depressive behavior, and may be involved in the predisposition of alcohol consumption (Vetter et al., 2002; Ryabinin et al., 2012). Ucn 2 may be linked to depression but not anxiety (Ryabinin et al., 2012). Ucn 2 appears to influence social behavior, including aggression (Breu et al., 2012), as mice deficient in Ucn 2 are less aggressive and prefer passive social interaction. Finally, Ucn 1 and CRF receptors have been found in the auditory system (Graham et al., 2010) and the peptide appears to be involved in the development and maintenance of hearing (Vetter et al., 2002).

Urocortin 3 is the latest addition of the CRF family of peptides, initially identified in the brains of humans and rodents (Hsu and Hsueh, 2001; Lewis et al., 2001). Sequence analyses show that Ucn 3 is more closely related to Ucn 2 than Ucn 1 or CRF. Human and mouse Ucn 3 share 40% homology with human and mouse Ucn 2, but only 21 and 18%, respectively, with human and mouse Ucn 1 and 32 and 26% with CRF (Lewis et al., 2001). Accumulating evidence, as discussed below, has suggested that Ucn 3 is a critical regulator in energy homeostasis.

## CORTICOTROPHIN-RELEASING FACTOR RECEPTORS

Two receptors have been identified for CRF: type 1 and type 2 CRF receptors (CRF_1_ and CRF_2_), and amino acid sequence analysis has shown that the two receptors share approximately 70% homology (Perrin and Vale, 1999; Bale and Vale, 2004). Both CRF_1_ and CRF_2_ are G-protein-coupled receptors with seven transmembrane domains. These receptors signal predominantly through increased cAMP production, but additional signaling pathways including Ca^2^^+^, mitogen-activated protein kinase (MAPK), phospholipase C, protein kinase B, and ion channels have also been shown to couple to CRFRs (Kiang, 1997; Grammatopoulos, 2000; Brar et al., 2002). The two receptors differ significantly in their binding affinity for CRF peptides and anatomical distribution within the central nervous system (Chalmers et al., 1995; Van Pett et al., 2000; Hsu and Hsueh, 2001; Lewis et al., 2001). Biochemical studies have shown that CRF binds CRF_1_ with high affinity while showing modest affinity for CRF_2_ (Bale and Vale, 2004). Ucn 1 binds both CRF_1_ and CRF_2_ with equally high affinity while Ucn 2 and Ucn 3 demonstrate preferential specificity for CRF_2_. However, while Ucn 2 may bind and stimulate CRF_1_ at high, pharmacological concentrations (Reyes et al., 2001), Ucn 3 is highly selective to CRF_2_ and displays minimal affinity for CRF_1_ (Hsu and Hsueh, 2001; Lewis et al., 2001).

A circulating protein has been identified that binds CRF. It has been suggested that the function of CRF binding protein (CRFBP) is mainly to sequester CRF to reduce its HPA axis stimulation. Levels of CRFBP are elevated in pregnancy to dampen the negative effect of stress responses on the developing fetus (Goland, 1986). In addition to CRFBP, a splice variant of CRF_2_ has been identified that contains only the extracellular domain of the receptor and shown to circulate, bind, and sequester CRF as well (Bon et al., 1997; Chen et al., 2005). This splice variant also binds Ucn 1 but has very low affinity for Ucn 2 and 3 (Chen et al., 2005).

Anatomical mapping studies have demonstrated that CRF_1_ and CRF_2_ have distinct distributions in the brain. CRF_1_ has a wide distribution throughout the brain with high density in the medial septal area, amygdala, and cerebellum (Chalmers et al., 1995; Van Pett et al., 2000). CRF_2_ has three major variants that differ in their N-terminal domains: CRF_2(a)_, CRF_2(b)_, and CRF_2(c)_. In murine brains, CRF_2(a)_ is predominantly expressed in the hypothalamus, lateral septum (LS), and dorsal raphe (Chalmers et al., 1995; Van Pett et al., 2000). CRF_2(b)_ is found predominantly in the periphery, including in skeletal muscle, the gastrointestinal tract, and the heart (Kanno, 1999; Wiley and Davenport, 2004; Porcher et al., 2005; Tache and Bonaz, 2007). CRF_2(c)_ is found only in the human brain (Kostich et al., 1998).

The function of CRF_1_ has been closely associated with the stress response, including the release of adrenocorticotropic hormone (ACTH) from the anterior pituitary and behavioral adaptation to stressors (Bale and Vale, 2004). The physiological role of CRF_2_, however, is less defined. Functional studies have shown this receptor is involved in an array of homeostatic regulations, with most of its actions regulating energy balance by modulating feeding, blood glucose levels, and energy expenditure. This review will focus on recent advances in the understanding of the physiological role of CRF_2_ and Ucn 3 in the brain, particularly in the hypothalamus, in the regulation of energy balance.

## CRF_2_ IN ENERGY HOMEOSTASIS

Loss-of-function studies with CRF_2_ null mice and pharmacological studies with CRF_2_ agonists both suggest that endogenous CRF_2_ plays a physiological role in energy balance. Evidence from several studies that examined ingestive behavior of CRF_2_ null mice showed that endogenous CRF_2_ is required for the anorectic effect of CRF peptide and is involved in control of the meal size during active phase of eating and following acute exposure to the stress (Bale et al., 2000; Coste et al., 2000; Pelleymounter et al., 2000; Tabarin et al., 2007). Similarly, infusion of antisense oligonucleotides to CRF_2_ mRNA attenuates both CRF and Ucn 1-induced hypophagia and corticosterone secretion (Smagin et al., 1998). Furthermore, CRF_2_ deletion protects mice from high-fat diet-induced insulin resistance and glucose intolerance (Bale et al., 2003). Taken together, it is clear that CRF_2_ is responsible for mediating the effect of CRF family peptides on the suppression of feeding and is involved in corticosterone secretion and glucose homeostasis.

In addition to feeding, CRF_2_ is involved in regulating energy expenditure. CRF_2_ knockout (KO) mice have more active metabolism and lose heat faster than wildtype (WT) mice (Carlin et al., 2006), suggesting an exaggerated level of sympathetic activity. Moreover, CRF_2_ KO mice have higher brown adipose tissue (BAT) temperature and, when given a choice between room temperature and warm areas, prefer warmer areas more than WT mice (Carlin et al., 2006). The KO mice also have higher oxygen consumption and carbon dioxide production and reduced respiratory exchange rate (Carlin et al., 2006), indicating a preference in fatty acid oxidation over carbohydrate utilization in the KO mice. It was suggested that a lack of functional CRF_2_ leads to elevated CRF_1_ activity, which consequently increases sympathetic nervous system (SNS) activity to promote lipolysis (Carlin et al., 2006). This hypothesis appears to disagree with pharmacological studies, as activation of central CRF_2_ (discussed below), in most cases, results in increased SNS activity. This apparent discrepancy may result from a number of possibilities including compensatory mechanisms due to total body KO of the receptor as compared to acute, local stimulation of the receptor in the brain. Obviously more studies are needed to further elucidate this issue.

Leptin, a hormone secreted by adipocytes, is a potent appetite suppressant (Uehara et al., 1998; Zigman, 2003). A number of studies suggest that its effect on feeding may involve the CRF receptor system. Though leptin treatment greatly decreases food intake, when co-administered with a non-selective CRF receptor antagonist, food intake remains at a nearly normal level (Gardner et al., 1998), suggesting the CRF system is a downstream target of leptin in the brain. However, this notion was recently challenged by a study (Harris, 2010) demonstrating that CRF_2_ is not essential for the effects of leptin on energy balance, including feeding and body weight regulation. Again this discrepancy may be due to the nature of global CRF_2_ KO, which potentially results in functional compensation such as exaggerated CRF_1_ activity in these mice (Carlin et al., 2006). Therefore, central or specific brain area deletions of CRF_2_ may be necessary to further evaluate the interaction of leptin and the CRF system in the brain.

## UROCORTIN 3

### ANATOMICAL LOCATION OF Ucn 3

Urocortin 3 is found both in the periphery and in the brain. In the periphery, it is expressed in the digestive tract, muscle, thyroid and adrenal glands, pancreas, heart, spleen, and skin (Hsu and Hsueh, 2001; Lewis et al., 2001). In the brain, neurons expressing Ucn 3 are concentrated in the medial amygdala (MeA) and hypothalamus (Lewis et al., 2001; Li et al., 2002). In the hypothalamus, the major Ucn 3 cell population is near the rostral perifornical hypothalamic area (rPFA; Li et al., 2002). Specifically, Ucn 3-positive cells are gathered around the fornix lateral to the paraventricular nucleus of the hypothalamus (PVH). This group extends rostrally and stays close to the fornix into the posterior part of the bed nucleus of the stria terminalis (pBNST) and medially into the anterior parvicellular part of the PVH (PVHap). A recent study has elucidated anatomical heterogeneity within this hypothalamic Ucn 3 cell population, as neurons of the rostral part (PVHap/pBNST) project to the ventromedial hypothalamus (VMH), and those of the caudal part, residing in the rostral perifornical hypothalamus (rPFH), projects to the LS (Chen et al., 2011). A second group of Ucn 3-positive cells is found in the median preoptic nucleus (MnPO; Li et al., 2002). In the forebrain, prominent Ucn 3 nerve fibers and terminals are found in the VMH, LS, MeA, and BNST (Li et al., 2002). These areas also express high levels of CRF_2_ (Chalmers et al., 1995; Van Pett et al., 2000). This overlap of Ucn 3 and CRF_2_ distribution and the high affinity of the peptide for the receptor strongly suggest that Ucn 3 is an endogenous ligand for CRF_2_ in these brain areas.

### METABOLIC EFFECTS OF Ucn 3

All 3 Ucns bind CRF_2_ and may each be responsible for some of the receptor’s energy homeostatic effects. However, Ucn 3 alone has myriad metabolic effects.

#### Feeding

Central administration, KO, and overexpression studies reveal a role of Ucn 3 in the regulation of feeding. When directly infused into the lateral ventricles, Ucn 3 decreases nocturnal food and water intake in a dose dependent manner, primarily due to decreased meal frequency, and this effect was eliminated with concomitant CRF_2_ antagonist treatment (Fekete et al., 2007). The anorectic effect of Ucn 3 is not due to distaste for food, as no concurrent taste aversion develops (Fekete et al., 2007). Consistent with pharmacological evidence, genetic Ucn 3 deficiency appears to lead to overeating. Though Ucn 3 KO mice have similar body weights to WT animals, KO mice eat more and have increased accumulated food intake. Similar to CRF_2_ null mice, Ucn 3 KO mice exhibit elevated nocturnal feeding, when greatest spontaneous food intake naturally occurs (Chao et al., 2012). Taken together, it is clear that Ucn 3 in the brain functions as a potent anorectic agent. However, a study that used genetic overexpression of Ucn 3 challenged this notion. Under regular chow-fed condition, mice with overexpression of Ucn 3 (*Ucn3+*) have higher body mass-adjusted food intake than WT and are heavier than WT controls due to increased lean body mass (Jamieson et al., 2011). On the other hand, *Ucn3+* mice do not gain as much weight as WT mice when fed with a high-fat diet (Jamieson et al., 2011). This result seems to be in line with the notion of Ucn 3 as an anorectic agent. The discrepancy between different mouse models can be due to a number of possibilities including the ramification of Ucn 3 overexpression both in the brain as well as in the periphery.

A temporally and spatially controlled viral approach to overexpress Ucn 3 in the rPFH shows that Ucn 3 in the rPFH does not modulate food intake (Kuperman et al., 2010), as mice with Ucn 3 overexpression in the rPFH ingest consume similar amount of food as control mice (Kuperman et al., 2010). The rPFH-specific Ucn 3-overexpressing mice show a trend toward being heavier, but retain the same fat–lean mass percentages as control mice (Kuperman et al., 2010). As mentioned above, Ucn 3 cells in the rPFH project mainly to the LS with minimal projection to the VMH. Therefore, it is reasonable to assume that CRF_2_ in the LS will be overstimulated in this mouse model. Interestingly, a number of studies have shown that CRF_2_ in the LS is involved in suppression of food intake (Wang and Kotz, 2002; Bakshi et al., 2007). Currently, it is unclear as to why overexpression of Ucn 3 in the rPFH fails to suppress feeding. The expression of CRF receptor has been shown to be subject to ligand-induced receptor down-regulation (Rabadan-Diehl et al., 1996; Eghbal-Ahmadi et al., 1997). It is conceivable that chronic elevated Ucn 3 input to the LS in Ucn 3 rPFH overexpression mice may lead to alteration in CRF_2_ expression and consequently reduced response to Ucn 3 stimulation in the LS. Thus, it is possible that acute stimulation of CRF_2_ in the LS suppresses feeding, but chronic stimulation of CRF_2_ in the LS in mice with Ucn 3 overexpression in the rPFH may lead to negative feedback to balance the effect of CRF_2_ in feeding. Clearly, more studies are needed to elucidate the effect of Ucn 3 overexpression in the regulation of food intake. In addition to Ucn 3, Ucn 1 neurons in the midbrain Edinger–Westphal nucleus have been shown to innervate the LS (Kozicz et al., 1998; Bittencourt et al., 1999). Therefore, both Ucn 1 and 3 may contribute to the effect of CRF_2_ on feeding in the LS.

The mechanism of Ucn 3-induced anorexia has not been directly studied. Central administration of CRF_2_ agonists, including Ucn 3, have been shown to inhibit gastric emptying (Martinez et al., 2004; Stengel and Tache, 2009), and elevate blood glucose levels (Jamieson et al., 2006; Chen et al., 2010). Both reduced gut motility and hyperglycemia have been shown to induce satiation and reduce feeding (Ritter, 2004; Cummings and Overduin, 2007; Wolfgang et al., 2007; Cha et al., 2008). Therefore, multiple mechanisms are potentially involved in mediating the anorectic effect of Ucn 3 in the brain.

#### Energy homeostasis

Similarly to CRF_2_, Ucn 3 is also involved in energy expenditure. Central administration of Ucn 3 increases motor activity (Ohata and Shibasaki, 2004). Similarly, transgenic Ucn 3 overexpression mice (*Ucn3+*) have an increased respiratory exchange ratio and increased motor activity in their home cages (Jamieson et al., 2011). Furthermore, mice with Ucn 3 overexpressed in the rPFH had an increased respiratory exchange ratio and elevated heat production (Kuperman et al., 2010). This is consistent with the notion that Ucn 3 is involved in SNS activity and energy homeostasis. Ucn 3 KO mice, on the other hand, do not show the same pattern; there are no differences in oxygen consumption, heat production, or activity levels (Chao et al., 2012). Therefore, KO of Ucn 3 in specific brain areas may provide better insight into the role of specific populations of Ucn 3 cells in the brain in energy expenditure.

#### Glucose homeostasis

Several studies have revealed a complex role of Ucn 3 in glucose homeostasis. Though adult Ucn 3 KO mice fed a chow diet show no differences in glucose tolerance and insulin sensitivity compared to WT mice (Li et al., 2007), Ucn 3 KOs have lower basal insulin levels and show a greater rebound in blood glucose levels after the initial hypoglycemia during an insulin tolerance test (Li et al., 2007; Chao et al., 2012). Furthermore, under high-fat diet feeding, adult KO mice are more metabolically resilient. The Ucn 3 KO mice have lower plasma insulin and blood glucose concentrations than WT mice, remain sensitive to insulin and do not develop glucose intolerance and liver steatosis with the same frequency of WT mice (Li et al., 2007). Moreover, aged KO mice show better glucose homeostasis than age-matched WT mice (Li et al., 2007). Overall, Ucn 3 deficiency appears to protect the mice from metabolic disorders caused by high-fat feeding. It is noteworthy that Ucn 3 is expressed in pancreatic β cells and has been shown to play a critical role as a local regulator in insulin secretion (Li et al., 2003). Thus, it is likely that Ucn 3 in both the brain and in the periphery, especially in the pancreas, contribute to the phenotypes observed in Ucn 3 null mice.

On the other hand, *Ucn3+* transgenic mice also appear to be protected against excessive metabolic challenge, having decreased fed and fasting blood glucose levels and increased tolerance to glucose when challenged in a glucose tolerance test (Jamieson et al., 2011). Fasting insulin levels in *Ucn3+* mice are also lower than that in WT mice, though an insulin tolerance test shows no differences in insulin sensitivity (Jamieson et al., 2011). When challenged with a high-fat diet, *Ucn3+* mice fare better than the WT littermates, maintaining normal body weight and low blood glucose levels, but display comparable insulin sensitivity to the WT control mice (Jamieson et al., 2011). Though *Ucn3+* mice show improved glucose homeostasis and insulin sensitivity, overexpression of Ucn 3 in the rPFH produces the opposite effect; rPFH Ucn 3-overexpressing mice show reduced insulin sensitivity and increases basal insulin levels, however they show no difference in glucose tolerance (Kuperman et al., 2010).

While genetic KO of Ucn 3 appears to have metabolic protective qualities, the effect of Ucn 3 overexpression is unclear. Full body overexpression of Ucn 3 seems protective, while targeted overexpression within the rPFH appears metabolically detrimental. Interestingly, *Ucn3+* mice have lower fasting blood glucose levels and higher energy intake under basal conditions (Jamieson et al., 2011). As stated earlier, CRF_2_ is expressed abundantly in a number of peripheral tissues including skeletal muscle (Perrin et al., 1995; Stenzel et al., 1995; Wiley and Davenport, 2004; Porcher et al., 2005; Kuperman et al., 2011). Stimulation of muscle CRF_2_ has been shown to promote thermogenesis (Solinas et al., 2006). Moreover, muscle CRF_2_ is involved in regulating skeletal muscle mass (Hinkle et al., 2003) and consistent with this notion, *Ucn3+* mice have increased muscle mass (Jamieson et al., 2011). Thus, it is conceivable that the improved glucose homeostasis of *Ucn3+* mice is due, at least in part, to stimulation of muscle CRF_2_ by ectopic overexpression of Ucn 3 in the periphery.

#### Thermoregulation

Functional studies have shown that Ucn 3 is involved in thermoregulation, potentially acting on brown fat. It was found that Ucn 3 induced a significant increase in body temperature, from 37.2 to 38.6°C (99.0 to 101.5°F), when injected into the lateral ventricles of rats (Telegdy et al., 2006). Temperature gradually decreased after peaking 2 h after Ucn 3 administration, but remained significantly elevated for a total of 4 h (Telegdy et al., 2006). Moreover, pretreating animals with CRF_2_ but not CRF_1_ antagonists completely blocked Ucn 3-induced hyperthermia, indicating that the pyrogenic action of Ucn 3 is mediated by CRF_2_ (Telegdy and Adamik, 2008). Noraminophenazone, a cyclooxygenase inhibitor, simultaneously applied with Ucn 3 also prevented the temperature increase and also attenuated the increase when administered 30 min after Ucn 3 treatment (Telegdy et al., 2006). This indicates that the arachidonic acid cascade forming prostaglandin is a downstream target of central Ucn 3 and CRF_2_ system in thermoregulation. Currently, the brain loci that may mediate the effect of Ucn 3 in body temperature remain elusive. The MnPO is known to be a center for thermoregulation and expresses high concentrations of the prostaglandin receptor EP3 (Morrison et al., 2008). It has been shown that prostaglandins play an important role in the MnPO through EP3 to regulate body temperature (Morrison et al., 2008). Furthermore, the presence of a group of Ucn 3 cells in the MnPO (Li et al., 2002) suggests that Ucn 3 might be involved in MnPO mediates pyrogenic effects.

### REGULATION OF Ucn 3 EXPRESSION IN THE BRAIN

The expression of Ucn 3 in the brain has been determined in a number of stress paradigms and metabolic challenges. It was found that restraint stress rapidly elevates Ucn 3 gene expression in the MeA and that the elevated Ucn 3 mRNA levels return to basal levels 4 h after the stress (Jamieson et al., 2006). Restraint stress also increases Ucn 3 mRNA levels in the rPFA but with a slower time course compared to that of the MeA (Venihaki et al., 2004; Jamieson et al., 2006). Adrenalectomy greatly elevates Ucn 3 expression in the rPFA, while corticosterone replacement returns the expression to a basal level (Jamieson et al., 2006). This result indicates that corticosterone may be involved in stress-mediated Ucn 3 gene expression in the rPFA. Hemorrhage decreases Ucn 3 expression in the MeA after 30 min, and 48 h of food deprivation also decreases Ucn 3 expression in the MeA (Jamieson et al., 2006).

**FIGURE 1:**
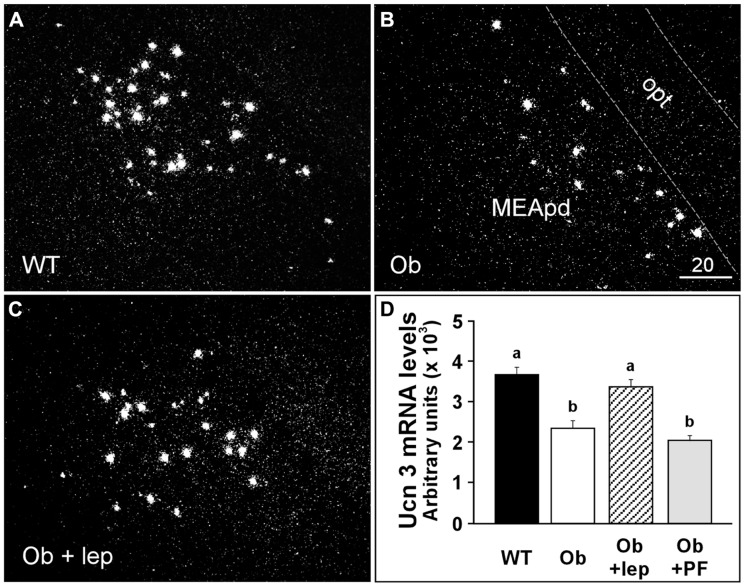
**Representative darkfield photomicrographs showing Ucn 3 mRNA expression (white clusters) in a wildtype mouse (A), an ob/ob obese mouse (B), and an ob/ob mouse treated with leptin (0.1 mg/kg/day for 2 days) (C)**. **(D)** Summary of Ucn 3 mRNA levels in the MeA of WT and Ob/ob obese mice treated with vehicle or leptin. The Ucn 3 mRNA levels in an additional group of Ob/ob mice that was paired-fed were also determined. Different letters represent statistical significance: *p* < 0.05. MeApd, medial nucleus of amygdala, posterodorsal part; opt, optic tract. Scale bar = 20 μm.

The expression of Ucn 3 has also been examined in genetically obese rodent models. Food deprivation increases Ucn 3 mRNA expression in the dorsal part of the medial amygdala (MeD) in obese Fa/Fa Zuker rats and has no effect on Ucn 3 expression in the rPFH (Poulin et al., 2012). In contrast, lean Fa/? rats show increased Ucn 3 expression in the rPFH but not the MeD after food deprivation (Poulin et al., 2012). Ucn 3 mRNA expression returns to normal after 24 h of refeeding (Poulin et al., 2012). In ob/ob obese mice, Ucn 3 expression is significantly reduced in the MeA (**Figure 1**; Li and Vale, 2002), and leptin treatment reverses Ucn 3 expression in this area. Interestingly, pair-feeding in ob/ob mice failed to modulate Ucn 3 expression in the MeA. These results indicate that Ucn 3 expression in the MeA is regulated by leptin. Taken together, these studies further support the notion that endogenous Ucn 3 in the brain is sensitive to metabolic signals and energy status and potentially plays an important role in regulating energy homeostasis.

## THE VENTROMEDIAL HYPOTHALAMUS

The anatomical distribution of CRF_2_ in the brain has provided important insight into possible areas that mediate the effects of CRF_2_ in energy balance. The VMH has received significant attention due to its abundant expression of CRF_2_ and well-known role in regulating energy homeostasis, feeding, and blood glucose levels.

The VMH is parceled on cytoarchitectonic grounds into three major divisions: dorsomedial (VMHdm), central, and the ventrolateral (VMHvl) parts (Gurdjian, 1927). The VMH volume is larger in males compared with females, and this difference is largely accounted for by the VMHvl, which in females is significantly smaller than that in males (Dugger et al., 2007). Further, VMHvl in female rats express higher levels of estrogen receptors than that in males, and has been shown to play a critical role in regulating lordosis behavior in females (Flanagan-Cato, 2011) and aggressiveness in males (Lin et al., 2011).

The VMH has long been considered a critical brain area in the regulation of energy homeostasis. Lesion of the VMH results in hyperphagia, hyperinsulinemia, reduction of SNS activity, increase of fat mass, and reduction of energy expenditure that ultimately leads to storage of excess of energy and obesity (Bernardis and Frohman, 1971; Inoue et al., 1977; Cox and Powley, 1981; Niijima et al., 1984; Sakaguchi et al., 1988; Ruffin and Nicolaidis, 1999; King, 2006). Conversely, stimulation of the VMH results in predominately opposite phenotypes including induction of satiety, increase in SNS activity, lipolysis, and thermogenesis (King, 2006). In recent years, the importance of the VMH in energy homeostasis has been further ascertained with the aid of improved molecular tools and mouse genetics (Sternson et al., 2005; King, 2006; Chao et al., 2012). For example, mice with VMH-specific deletion of a number of genes including the leptin receptor (Dhillon et al., 2006; Bingham et al., 2008), estrogen receptor α (Musatov et al., 2006, 2007), and vesicular glutamate transporter-2 (VGLUT2; Tong et al., 2007) result in a number of abnormalities such as increased feeding, reduced energy expenditure, impaired glucose homeostatic regulation, and obesity. Moreover, mice bearing a deletion of steroidogenic factor 1 (SF1), a transcription factor involved in steroidogenesis that is highly enriched in the VMH, show similar phenotypes in energy homeostasis and are obese (Luo et al., 1994; Sadovsky et al., 1995; Shinoda et al., 1995).

### EXPRESSION OF CRF_2_ IN THE VMH

The VMH is one of the brain areas with prominent CRF_2_ expression (Chalmers et al., 1995; Van Pett et al., 2000). The expression is concentrated in the dorsomedial part of the nucleus with decreasing density toward the ventrolateral division of the VMH. The expression of CRF_2_ in the VMH has been shown to be sensitive to energy status and stress. For example, leptin injection increases and fasting decreases CRF_2_ expression in the VMH and ob/ob mice or Fa/Fa Zucker rats have lower CRF_2_ in the VMH compared to WT controls (Richard et al., 1996; Makino et al., 1998, 1999; Nishiyama et al., 1999). Furthermore, restraint stress and glucocorticoids increase CRF_2_ levels in the VMH (Makino et al., 1998, 1999). Thus, these data support the notion that CRF_2_ in the VMH is important in regulating energy balance.

**FIGURE 2:**
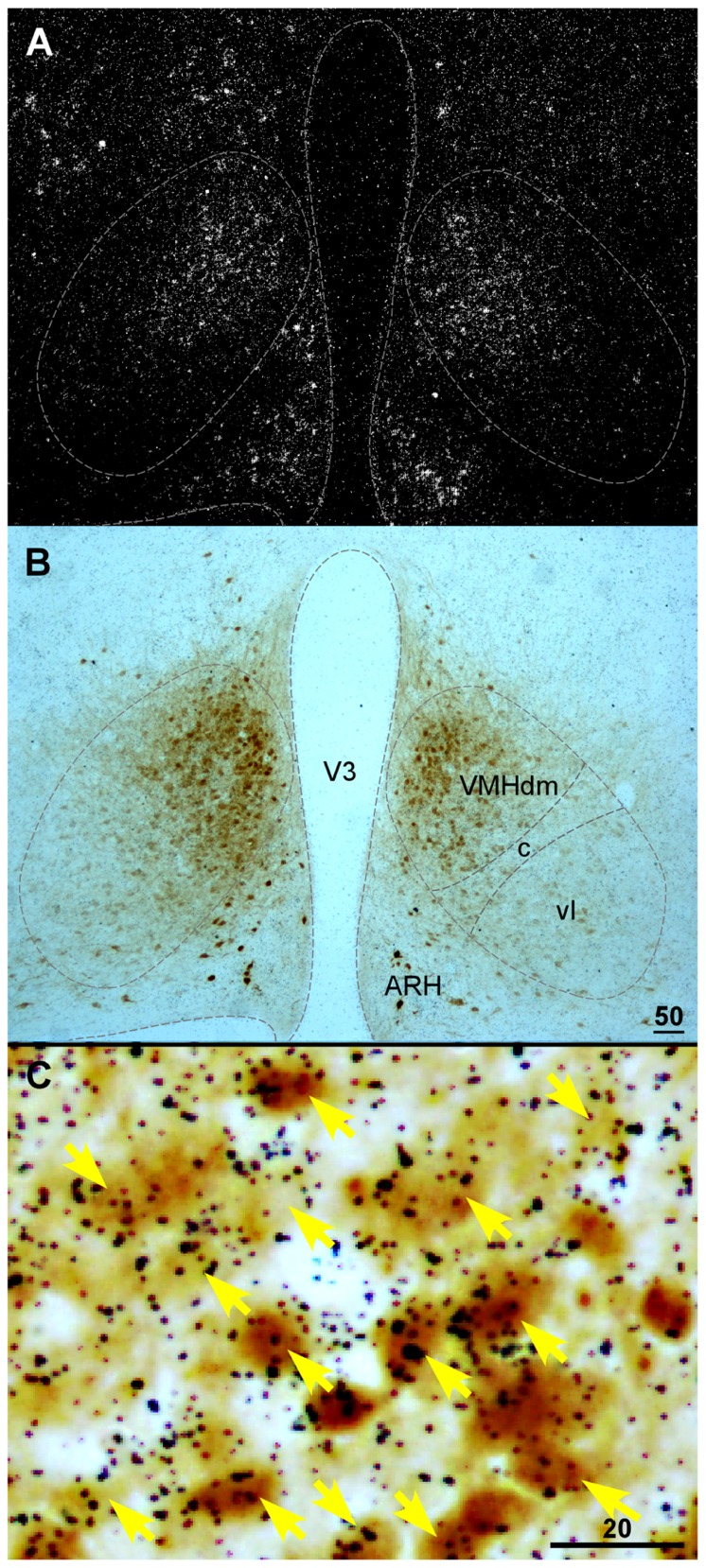
**(A)** Darkfield photomicrograph showing CRFR2 mRNA signal revealed by *in situ* hybridization in the basal hypothalamic area of a transgenic mouse expressing Cre recombinase (Cre) and enhanced yellow fluorescent protein (EYFP) in SF1-positive cells. Note that CRFR2 mRNA hybridization signal (white clusters) was abundant in the dorsomedial part of the VMH (VMHdm). **(B)** Bright field photomicrograph of the same area in **(A)** showing SF1-positive cells, revealed by immunostaining with anti-green fluorescent protein antibody (darkbrown precipitates) in the VMH. **(C)** High magnification of boxed area in **(A)** showing colocalization of CRFR2 (black dot clusters) and SF1 (brown precipitates) in the VMH. ARH, arcuate nucleus of hypothalamus; V3, third ventricle; VMHc, central part of the VMH; VMHdm, dorsomedial part of the VMH; VMHvl, ventrolateral part of the VMH. Scale bar = 50 μm **(B)**, 20 μm **(C)**.

Currently, detailed neurochemical phenotypes of CRF_2_-positive cells within the VMH remain unclear. Several proteins have been found to be expressed in the VMH including SF1, pituitary adenylate cyclase-activating polypeptide, the leptin receptor, and VGLUT2 (Fei et al., 1997; Elmquist et al., 1998; Ziegler et al., 2002; Segal et al., 2005; Kurrasch et al., 2007). It has been shown that CRF_2_ extensively colocalizes with VGLUT2 in the VMH (Chen et al., 2010). VGLUT2 mediates glutamate uptake into synaptic vesicles of excitatory neurons (Fremeau et al., 2001; Herzog et al., 2001; Takamori et al., 2001) and has been used extensively as a marker for excitatory glutamatergic neurons. The colocalization of CRF_2_ and VGLUT2 suggests that CRF_2_ is expressed predominately in excitatory neurons in the VMH. As mentioned above, SF1 is a nuclear receptor that regulates the transcription of key genes involved in sexual development and reproduction (Parker et al., 2002). In adults, SF1 expression is specifically confined to the VMH (Parker et al., 2002). In SF1 null mice, CRF_2_ mRNA expression is nearly undetectable in the VMH (Luo et al., 1994; Sadovsky et al., 1995; Shinoda et al., 1995), suggesting CRF_2_ is expressed in SF1 cells in the VMH. Consistent with this notion, it was found that more than 90% of CRF_2_ neurons in the dorsomedial part of the nucleus are also SF1-positive (**Figure 2**) with less colocalization of these two materials in the VMHvl (Digruccio et al., 2007).

### CRF LIGANDS INPUT INTO THE VMH

The anatomical distribution of a number of the CRF family peptides has been determined, and it was found that CRF and Ucn 1-expressing neurons provide minor innervation to the VMH (Swanson et al., 1983; Kozicz et al., 1998; Bittencourt et al., 1999). Interestingly, low levels of Ucn 1-immunoreactivity have been detected in cells in the VMH (Kozicz et al., 1998), suggesting Ucn 1 may be a local factor in the nucleus. Although Ucn 2 fiber distribution has not been determined, Ucn 2-positive cells have been found in a number of brain areas including the magnocellular part of the PVH, locus ceruleus, and facial motor nucleus (Reyes et al., 2001), and none of these areas provide extensive projection into the VMH (McBride and Sutin, 1977; Luiten and Room, 1980; Berk and Finkelstein, 1981; Kita and Oomura, 1982; Zaborszky, 1982; Fahrbach et al., 1989; Chen et al., 2011). Compared to other CRF innervations, Ucn 3 neuronal fibers abundantly innervate the VMH (Li et al., 2002). Similar to the expression of CRF_2_ in the VMH, Ucn 3-positive axonal fibers and terminals concentrate in the dorsomedial part of the VMH with reduced density toward the ventrolateral part of the nucleus (Li et al., 2002). As stated above, Ucn 3 neurons in the PVHap provide the major Ucn 3 afferent input into the VMH and Ucn 3 cells in the pBNST and the MeA comparatively provide moderate input into the nucleus (Chen et al., 2011). Interestingly, Ucn 3 cells in the rPFH, immediately caudal and adjacent to the PVHap, provide minimal Ucn 3 afferent input into the VMH and instead send strong innervation into the LS.

Neural inputs into the PVHap and MeA have been studied (Sawchenko and Swanson, 1983; Swanson and Petrovich, 1998; Uehara et al., 1998; Maras and Petrulis, 2010; Carrillo et al., 2011; Northcutt and Lonstein, 2011; Radley and Sawchenko, 2011; Van Hover et al., 2011). In general, the two brain areas receive similar afferent input from a number of brain regions including the septal nuclei and amygdala. However, a few subtle but significant exceptions should be noted. The PVHap receives prominent inputs from the hypothalamus, cortex, and brainstem, whereas the MeA receives strong inputs from the bed nucleus of the accessory olfactory tract and nucleus of stria medullaris. These findings suggest Ucn 3 cells in the PVHap receive inputs that transmit visceral and autonomic information while the Ucn 3 cell group in the MeA receives afferents that relay olfactory information. Thus, it is conceivable that central Ucn 3 may serve as a common peptide neurotransmitter in the various neural pathways that convey information from assorted neural signals into the VMH to coordinate the regulation of energy homeostasis.

### FUNCTION OF CRF_2_ IN THE VMH

When the function of CRF_2_ in the VMH was first assessed by injecting Ucn 1 into the VMH, it was found that the peptide potently suppresses food intake (Ohata et al., 2000). However, because CRF_1_ has been suggested to be expressed in the VMH (Cheng et al., 2007) and Ucn 1 has equally high affinity for both CRF_1_ and CRF_2_ (Vaughan et al., 1995), it remains possible that CRF_1_ may also contribute to the anorectic effect of Ucn 1 in the VMH. More recently, when the CRF_2_-selective ligand Ucn 3 was identified (Lewis et al., 2001), the function of CRF_2_ in the VMH was re-examined by site-specific injection of Ucn 3 into the VMH (Fekete et al., 2007; Chen et al., 2010). Consistent with the Ucn 1 injection study, stimulation of CRF_2_ by Ucn 3 in the VMH significantly suppresses feeding. Moreover, stimulation of CRF_2_ in other regions including the PVH, amygdala, and the lateral hypothalamus fails to modulate feeding (Ohata et al., 2000; Fekete et al., 2007; Chen et al., 2010), reinforcing the notion that VMH CRF_2_ plays a critical role in mediating the effect of CRF peptides in suppressing food intake.

In addition to appetite suppression, activation of CRF_2_ in the VMH results in rapid elevation of blood glucose levels (Chen et al., 2010). This is consistent with the function of VMH neurons in glucose homeostasis, as VMH neurons have been suggested to play an important role in regulating glucose levels via glucose sensing, and modulating glucose production in peripheral tissues (Kang et al., 2004; Levin et al., 2004). On the other hand, it has been shown that stimulation of CRF_2_ in the VMH suppresses insulin-induced release of glucagon and epinephrine (McCrimmon et al., 2006), indicating that VMH CRF_2_ exerts a negative control over the counterregulatory response (CRR). Taken together, it is possible that the functional role of CRF_2_ in the VMH in glucose homeostasis is context dependent. When blood glucose is low as a result of hyperinsulinemia, CRF_2_ in the VMH negatively regulates the CRR response. On the other hand, under normoglycemia, CRF_2_ induces acute hyperglycemia to facilitate fuel mobilization perhaps in response to stress. We have also found that CRF_2_-positive neurons in the VMH are sensitive to glucose, as high glucose inhibits and low glucose stimulates the neuronal activity (Digruccio et al., 2007).

Type 2 CRF receptor has been shown to modulate the HPA axis. For example, CRF_2_ KO mice display altered HPA hormonal secretion, and central Ucn 3 injection facilitates stress-induced ACTH secretion (Jamieson et al., 2006). On the other hand, activation of CRF_2_ in the VMH fails to modulate HPA hormone secretion (Chen et al., 2010), indicating that the receptor in the VMH is not essential for central Ucn 3-induced HPA activation. Thus, CRF_2_-positive brain loci that are important for modulation of the HPA axis remain to be determined.

### PHYSIOLOGICAL ROLE OF CRF_2_ IN THE VMH

To probe the physiological role of endogenous CRF_2_ in the VMH, VMH-specific CRF_2_ knockdown mice were generated by injection of a lentiviral vector expressing CRF_2_ small hairpin RNA (shRNA; Chao et al., 2012). Mice injected with CRF_2_ shRNA displayed significantly reduced CRF_2_ mRNA levels and gain more weight, mostly in white fat, than control mice. Furthermore, similar to Ucn 3 null mice, mice with reduced CRFR2 in the VMH exhibited elevated basal food intake and ate more than the control mice after overnight fasting. This result indicates that CRF_2_ in the VMH serves as a brake to facilitate the cessation of feeding. This study suggests that CRF_2_ in the VMH plays a critical role in mediating the effect of central Ucn 3 in energy balance.

In addition to elevated feeding, mice with decreased expression of CRF_2_ in the VMH display reduced lipolysis and increased adiposity in white fat (Chao et al., 2012). This is likely due to reduced SNS activity, as the VMH has been shown to regulate lipolysis via sympathetic outflow (Kumon et al., 1976; Takahashi and Shimazu, 1981; Ruffin and Nicolaidis, 1999). Interestingly, CRF_2_ knockdown in the VMH has no major impact in heat production or uncoupling protein 1 expression in BAT, suggesting that CRF_2_ in the VMH does not significantly modulate thermogenesis in BAT. This result appears to disagree with earlier reports that VMH is involved in regulating thermogenesis in BAT (Perkins et al., 1981; Kim et al., 2011). Anatomical studies have demonstrated a compartment-specific organization of innervation of different peripheral organs, as abdominal fat and subcutaneous fat are innervated by different neural pathways (Kreier et al., 2002, 2006). It is possible that CRF_2_-positive neurons are a subpopulation of cells in the VMH that regulate SNS outflow to white fat without significant functional impact on BAT. Heterogeneity of VMH neurons has been reported as overlapping but distinct subpopulation of neurons in the VMHvl that are critical in regulating fighting and mating (Lin et al., 2011).

Consistent with the function of CRF_2_ in regulating blood glucose levels, mice with reduced CRF_2_ expression in the VMH show improved glucose homeostasis compared to control mice (Chao et al., 2012). Moreover, mice with CRF_2_ knocked down in the VMH exhibit an exaggerated rebound in blood glucose levels compared to control mice after the initial hypoglycemic response to insulin challenge. This result agrees with the study by McCrimmon et al. (2006), who found that injection of Ucn 3 into the VMH suppresses the hypoglycemia-induced CRR response. Taken together, these studies strongly argue that CRF_2_ is a critical molecular mediator in VMH regulation of glucose homeostasis.

### NEUROCIRCUITS UNDERLYING THE EFFECT OF VMH

To understand how VMH CRF_2_ neurons regulate output functions and to describe an anatomical link between these neurons and the SNS, it is necessary to determine their axonal projections to identify downstream targets in the brain. A number of anterograde tracing studies utilizing different tracers have been performed to evaluate the projections of VMH neurons. Generally, it was found that VMH neurons project extensively to neighboring hypothalamic nuclei including the anterior and paraventricular nucleus, BNST, and periaqueductal gray (PAG; Saper et al., 1976; Canteras et al., 1994). These anatomical studies raise an interesting dilemma. Although it is clear that VMH activity modulates SNS activity, these studies failed to observe direct VMH efferents within well-known autonomic centers in the brainstem. Thus, it was concluded that the VMH likely modulates SNS activity indirectly by first projecting to a relay center such as the PAG.

**FIGURE 3:**
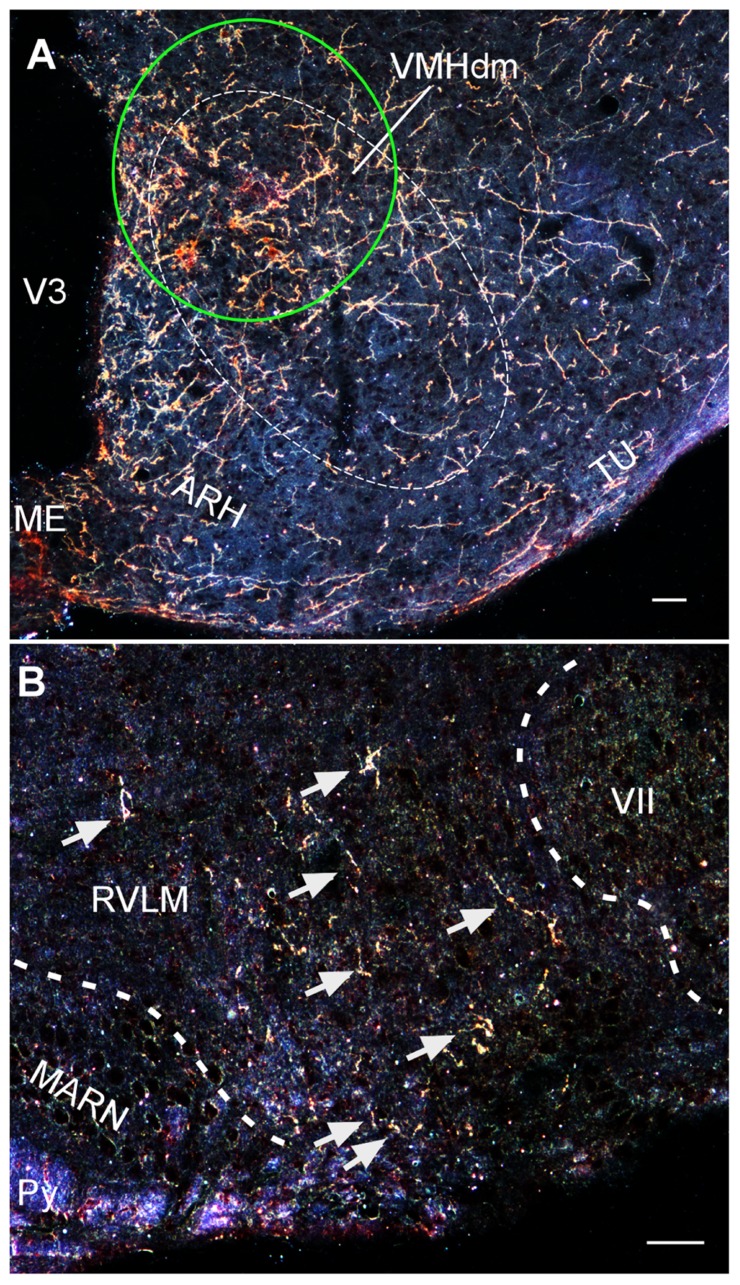
**(A)** Darkfield photomicrograph showing the injection site (green circle) of an adenoviral vector encoding Cre-regulated expression of farnesylated green fluorescent protein (GFP_f_) delivered into the VMH of a CRFR2-Cre mouse. The expression of GFP_f_ is normally silenced due to a Cre-regulated transcription block sequence inserted upstream of GFP_f_ reporter cassette. In the presence of Cre, the transcription block sequence is removed to allow GFP_f_ to be expressed in Cre-positive cells. **(B)** Darkfield photomicrograph depicting the distribution of VMH CRFR2-positive fibers (golden staining) within the rostral ventrolateral medulla (RVLM). ARH, arcuate nucleus of hypothalamus; MARN, magnocellular reticular nucleus; ME, median eminence; Py, pyramidal tract; Tu, tuberal nucleus; V3, third ventricle; VII, facial nucleus; VMHdn, dorsomedial part of the ventromedial nucleus of hypothalamus. Scale bar = 50 μm.

Recently, using a conditional viral tracing approach, we have found that VMH neurons project to a number of important brainstem autonomic centers including the parabrachial nucleus, C1 catecholaminergic cell group in the rostral ventrolateral medulla, and the nucleus of solitary tract (Lindberg et al., 2011). Moreover, we have used the same approach to find that CRF_2_-positive cells in the VMH show similar axonal projections to these brainstem areas (**Figure 3**; Lindberg et al., 2011). These studies demonstrate that VMH neurons, including cells that express CRF_2_, can potentially modulate SNS activity by direct projections to brainstem autonomic centers.

## CONCLUSION

The function of CRF peptides and their receptors in coordinating hormonal, neuronal, and behavioral responses to stress is well recognized. Pharmacological studies have determined that the CRF_2_ receptors are involved in the regulation of energy homeostasis. Recent studies using various genetic mouse models and molecular tools have further ascertained the critical role of CRF_2_ and its selective ligands, including Ucn 3, in feeding, blood glucose regulation, SNS output, and peripheral metabolism. Moreover, CRF_2_ in the VMH mediates most, if not all, of the effects of central Ucn 3 on energy homeostasis.

It is clear that conflicting results have been observed between whole body and region- or tissue-specific KO or overexpression mouse models. Furthermore, several studies have demonstrated that anatomical or even functional heterogeneity exists within a seemingly single Ucn 3 cell population in the hypothalamus. Thus, a more detailed understanding of the physiological function of CRF_2_ and its selective ligands in the brain will be aided by brain region-specific transgenic animal models permitting manipulation of ligand or receptor expression. Study of such models will provide insight into the specific roles of CRF_2_ in modulating metabolic functions.

Regulation of energy balance under diverse challenges including stress, starvation, or high-fat diet requires numerous adaptive mechanisms in both central and peripheral tissues. It is now clear that central CRF_2_ and Ucn 3 are involved in this regulation, as the expression of the receptor and ligands are closely regulated under these challenges. It is thus conceivable that dysregulated CRF_2_ or ligand function potentially cause or exacerbate metabolic perturbations. This hypothesis can be easily tested with the above mentioned rodent models to determine the functional role of the CRF_2_ system in metabolic diseases. Moreover, a better understanding of the molecular mechanisms by which various stressors or metabolic signals regulate the expression and/or function of CRF_2_ and its ligand will provide significant insight into the potential role of the CRF family and its receptors in the pathophysiology of metabolic disorders including obesity and diabetes.

## Conflict of Interest Statement

The authors declare that the research was conducted in the absence of any commercial or financial relationships that could be construed as a potential conflict of interest.
